# Chlorogenic Acid Relieves the Lupus Erythematosus-like Skin Lesions and Arthritis in MRL/lpr Mice

**DOI:** 10.3390/ph15111327

**Published:** 2022-10-27

**Authors:** Ruxuan Wang, Xiaoyi Yang, Shen You, Mengyao Hao, Jianguang Li, Xiaoguang Chen, Jing Jin

**Affiliations:** 1 State Key Laboratory of Bioactive Substance and Function of Natural Medicines, Institute of Materia Medica, Chinese Academy of Medical Sciences & Peking Union Medical College, Beijing 100050, China; 2 College of Pharmacy, Xinjiang Medical University, Urumqi 830054, China; 3 College of Pharmacy, Xinjiang University of Science and Technology, Korla 841000, China

**Keywords:** chlorogenic acid, natural product, systemic lupus erythematosus, MRL/lpr, IL-17

## Abstract

Chlorogenic acid (CGA) is a phenylpropyl substance synthesized through the shikimic acid pathway. In addition to its anti-tumor, anti-inflammatory, and antioxidant abilities, CGA also has immunomodulatory effects. The aim of the present study is to investigate the therapeutic effects of CGA on the skin damage and arthritis caused by systemic lupus erythematosus (SLE) in an MRL/lpr mouse model. In the SLE model, female MRL/lpr mice at the age of 10 weeks old were treated with CGA daily or cyclophosphamide (CTX) weekly via intraperitoneal injection for three months. After treatment, CGA can significantly alleviate the skin and mucous membrane damage caused by SLE and has a certain improvement effect on arthritis. CGA could inhibit dsDNA expression to a certain extent but has no obvious regulation on ANA concentration. The ELISA and BioMAP results indicated that CGA might play an anti-inflammatory role by down-regulating the interleukin (IL)-17 level. In conclusion, our study demonstrates that CGA can alleviate multiorgan damage in MRL/lpr mice by reducing IL-17.

## 1. Introduction

Systemic lupus erythematosus (SLE) is an autoimmune disease characterized by the aberrant activity of the immune system and the overproduction of autoantibodies, leading to multisystem damage [1,2]. The pathogenesis of SLE remains identified; several mechanisms lead to autoimmunity and organ dysfunction, including genetic susceptibility, epigenetic modification, hormones, and environmental triggers [3]. The deposition of immune complexes composed of endogenous antigens and autoantibodies and a multitude of functional disturbances of B cells, T cells, and other antigen-presenting cells plays a critical role in the occurrence and development of SLE [4,5]. The incidence and prevalence of SLE varies greatly in regions and populations, with a global prevalence of 13–7713.5 per 100,000 individuals [6]. Women are typically pre-disposed to this condition, especially at childbearing age.

The treatment strategy of SLE focuses on reducing disease activity and preventing organ damage. The recommended treatments include antimalarial drugs, steroids, non-steroidal anti-inflammatory drugs, and immunosuppressants with methotrexate and cyclophosphamide (CTX) [7]. Therapeutics such as steroids and immunosuppressants are associated with substantial toxic effects and can contribute to the damage. This prompts the need to find new treatment modalities to improve efficacy and reduce the side effects of drug toxicity.

Chlorogenic acid (CGA) is a polyphenol widely present in nature and the human diet. Recently, CGA has been shown to have a variety of biological activities, such as those which are neuro-protective, hepatoprotective, anti-inflammatory, anti-oxidant, glucose and lipid metabolism regulatory, anti-tumor, and immune regulatory [8]. Studies have reported that CGA can modulate the tumor microenvironment and inhibit the growth of glioblastoma through the promotion of the polarization of tumor-associated macrophages from the M2 to the M1 phenotype [9,10]. Additionally, CGA can induce the expression of nitric oxide synthase, tumor necrosis factor-2 (TNF-α), and macrophage inflammatory protein-2 (MIP-2), which could help the body correctly recognize tumor cells by regulating the immune system. Furthermore, it could prevent the carcinogenesis of cells [11]. However, CGA has rarely been reported in autoimmune diseases. In this study, we aimed to explore the efficacy and intervention mechanism of CGA in SLE, in order to find better drugs for the treatment of SLE.

## 2. Results

### 2.1. CGA Relieves Incidence of Skin Damage in MRL/lpr Mice

**Figure 1:**
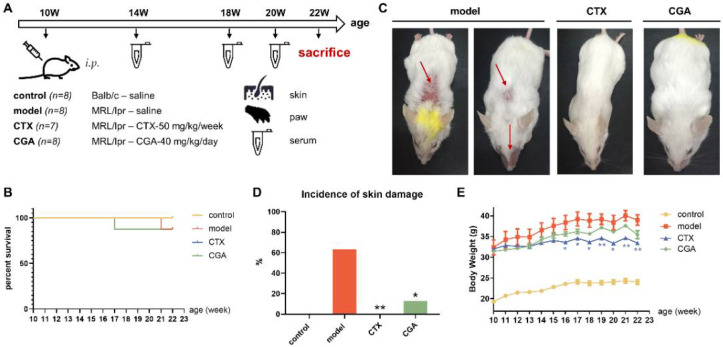
Ameliorative effect of chlorogenic acid (CGA) on skin damage in MRL/lpr mice. (**A**) Schematic of experimental design. One mouse in the model group died at 21 weeks of age while one mouse in the CGA group died at 17 weeks of age (**B**), which may be related to the disease process. (**C**) Photos of the entire body of MRL/lpr mice at 22 weeks. Mice in the model group showed skin damage to the back, nose, and mouth. (**D**) Incidence of skin damage at 3 months. (**E**) Body weights from 10 to 22 weeks. Control group: *n* = 8; model group: *n* = 8; cyclophosphamide (CTX) group: *n* = 7; CGA group: *n* = 8. Changes were tested using the chi-squared statistic from SPSS 13.0. * *p* < 0.05, ** *p* < 0.01 vs. model.

Skin damage is one of the most common clinical symptoms of SLE [12]; thus, we first evaluated the skin changes of MRL/lpr mice, a well-known model that is similar to human SLE. We used Balb/c mice as the control group. To determine the therapeutic potential, 40 mg/kg CGA was administered to 10-week-old female MRL/lpr mice with a daily intraperitoneal (i.p.) injection for 12 weeks. CTX (50 mg/kg), as the positive drug, was administered weekly with an i.p. injection. The schedule of this progress is shown in Figure 1A, and the survival curve is shown in Figure 1B. The skin lesions were obvious in the model group, and CGA and CTX may have some ameliorating effects. As shown in Figure 1C,D, the incidence rate of skin damage in the MRL/lpr mice reached 63% compared with that of the Balb/c mice, including damage to the eyes, nose, and back. Both CGA and CTX were able to inhibit lupus skin mucosal damage. CGA could significantly alleviate skin symptoms, and the skin damage rate was reduced to 13%. The effect of CGA is comparable to that of hydroxychloroquine, another SLE treatment drug [13]. CTX had a better therapeutic effect and none of the mice appeared to have skin damage. However, CTX could have caused significant weight loss in the animals from the 6th week of administration (Figure 1E), suggesting that CTX may have some toxicity.

### 2.2. CGA Ameliorates Epidermal Layer Damage and Dermal Mast Cells in MRL/lpr Mice

**Figure 2:**
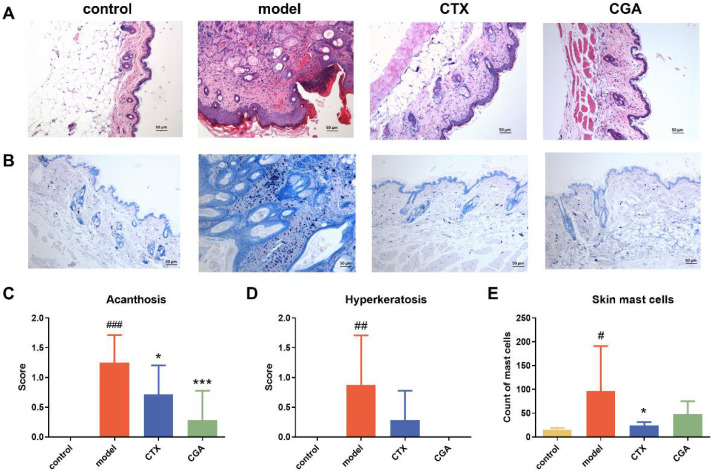
CGA treatment suppressed skin disease in MRL/lpr mice. HE staining (×100) (**A**) and toluidine blue staining (×100) (**B**) of the skin tissue treated with CGA and CTX. (**C**,**D**) The pathological score of acanthosis and hypertrophy. (**E**) Mast cells detected by toluidine blue in the affected skin of mice were counted. Control group: *n* = 8; model group: *n* = 8; CTX group: *n* = 7; CGA group: *n* = 7. The values are expressed as the mean ± SD. Changes were tested using the chi-squared statistic from SPSS 13.0. # *p* < 0.05, ## *p* < 0.01, ### *p* < 0.001 vs. control. * *p* < 0.05, *** *p* < 0.001 vs. model.

We examined the pathological changes of the skin tissue from MRL/lpr mice treated with CGA and CTX. For functional measurements, all the groups were sacrificed at 22 weeks of age. Histologically, acanthosis and hyperkeratosis of the epidermal layer were observed in the model group, while CGA and CTX significantly reduced the incidence as well as the pathological score (Figure 2A,C,D). Mast cells widely distributed around microvessels in the skin and the submucosa of internal organs could secrete a variety of cytokines and participate in immune regulation. Toluidine blue staining revealed that dermal mast cells were significantly increased in the model group. As expected, CGA and CTX caused a decrease in the number of dermal mast cells in MRL/lpr mice (Figure 2B,E). The significant improvement of lupus skin damage by CGA occurred mainly in the epidermis, which is consistent with the clinical manifestation of “improved thickness, hardness and reduced scaling”, while CGA reduced the number of mast cells in the dermis (Table 1).

### 2.3. CGA Treatment Alleviates Lupus-like Arthritis in MRL/lpr Mice

**Figure 3:**
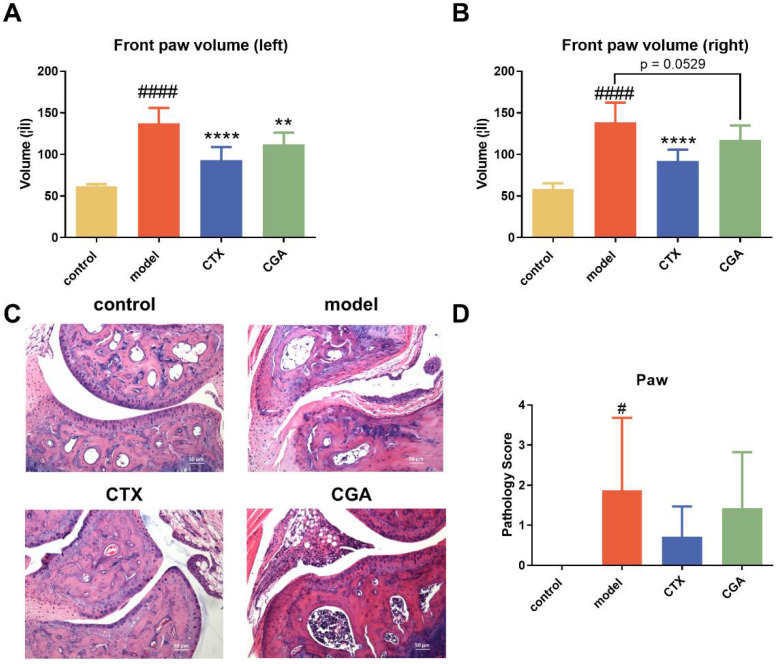
CGA treatment alleviated lupus-like arthritis in MRL/lpr mice. We evaluated the effect through front paw volume of left (**A**) and right (**B**), HE staining × 100 (**C**), and pathology score (**D**). Control group: *n* = 8; model group: *n* = 7; CTX group: *n* = 7; CGA group: *n* = 8. The values are expressed as the mean ± SD. Data were analyzed using one-way ANOVA test. # *p* < 0.05, #### *p* < 0.0001 vs. control, ** *p* < 0.01, **** *p* < 0.0001 vs. model.

Arthropathy is also a common manifestation of SLE patients. In this study, we evaluated the effects of CGA treatment on plantar swelling. Both CGA and CTX showed significant improvement in foot and plantar swelling in lupus arthritis (Figure 3A,B). The joint lesions of some animals in the model group showed infiltration of inflammatory cells in the joint capsule, cartilage erosion, synovial epithelial cell proliferation, and infiltration of synovial inflammatory cells (Figure 3C). Both CGA and CTX could reduce the lesions to a certain extent (Figure 3D).

### 2.4. CGA Decreased Serum Anti-dsDNA Antibody Concentration

**Figure 4:**
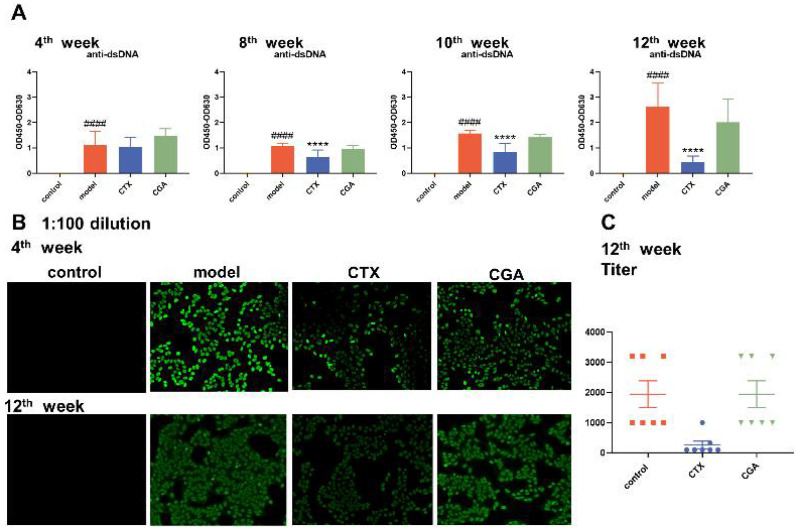
Changes in SLE-specific indicators of dsDNA (**A**) and antinuclear antibodies (ANA) (**B**,**C**). CTX significantly reduced serum dsDNA levels and ANA titers in a time-dependent manner, while CGA had a tendency to reduce dsDNA but did not significantly change the ANA titers. Fourth week: control group: *n* = 8; model group: *n* = 8; CTX group: *n* = 7; CGA group: *n* = 8. Eighth and tenth week: control group: *n* = 8; model group: *n* = 8; CTX group: *n* = 7; CGA group: *n* = 7. Twelfth week: control group: *n* = 8; model group: *n* = 7; CTX group: *n* = 7; CGA group: *n* = 7. #### *p* < 0.0001 vs. control, **** *p* < 0.0001 vs. model. The values are expressed as the mean ± SD. The ordinary one-way ANOVA was used to compare the values between groups.

The level of anti-dsDNA antibodies in the serum is a critical sign of the development of organ damage in SLE patients. We collected 4th week, 8th week, 10th week, and 12th week serum from the treated mice and performed enzyme-linked immunosorbent assay (ELISA) for anti-dsDNA antibodies. CTX significantly reduced the serum dsDNA levels in a time-dependent manner, and there was a tendency for CGA to reduce dsDNA, although no significant difference was noted (Figure 4A). Antinuclear antibodies (ANAs) are also key biomarkers in the evaluation of SLE. After 4 weeks of administration, all the animals except the Balb/c group were ANA positive with karyotype homogeneity showing SLE, with the same result at the 12th week (Figure 4B). The data showed that CTX could significantly reduce ANA drops. However, CGA did not change the titer of ANA (Figure 4C).

### 2.5. CGA Rebalances Serum Inflammatory Cytokines

**Figure 5:**
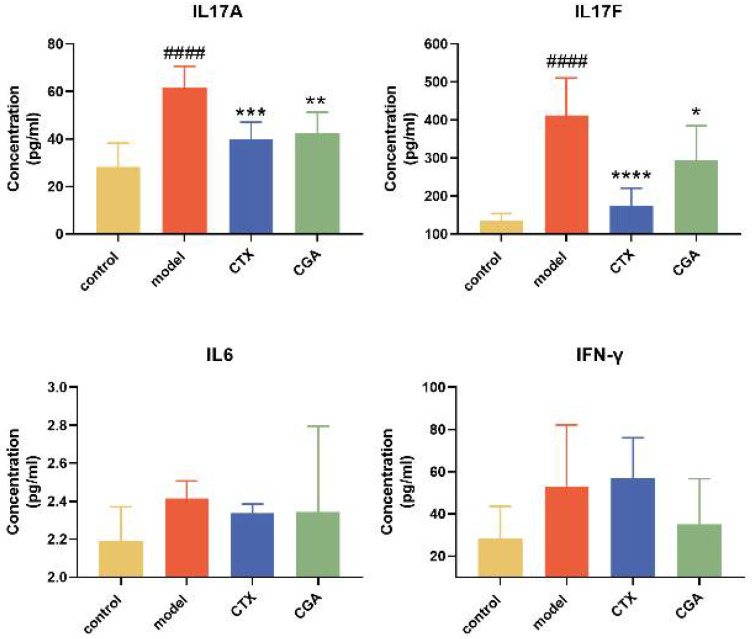
CGA decreases inflammatory cytokines levels in MRL/lpr mice. Serum concentrations of interleukin (IL)-17A, IL-17F, IL-6, and interferon-γ (IFN-γ) were measured in Balb/c and MRL/lpr mice at 22 weeks of age (mean ± SD, *n* = 7–8/group). Mice were treated with CTX and CGA for 12 weeks. Control group: *n* = 8; model group: *n* = 7; CTX group: *n* = 7; CGA group: *n* = 7. #### *p* < 0.0001 vs. control, * *p* < 0.05, ** *p* < 0.01, *** *p* < 0.001, **** *p* < 0.0001 vs. model.

In order to explore how CGA interferes with the progression of SLE, we collected serum for cytokine content detection after the treatment of MRL/lpr mice with CGA and CTX for 12 weeks. The results are shown in Figure 5. CGA could effectively reduce the levels of serum interleukin (IL)-17A and IL-17F, and it also had a tendency to reduce interferon-γ (IFN-γ) and IL-6 compared to the model group to a certain extent. At the same time, we also tested the changes in serum immunoglobulin A (IgA), immunoglobulin M (IgM), immunoglobulin G (IgG), the B cell activating factor of the TNF family (BAFF), and complement 3 (C3) (Figure S1) and found that CGA had no significant effect on these cytokines.

### 2.6. Effect of CGA on Human Primary Cell

**Figure 6:**
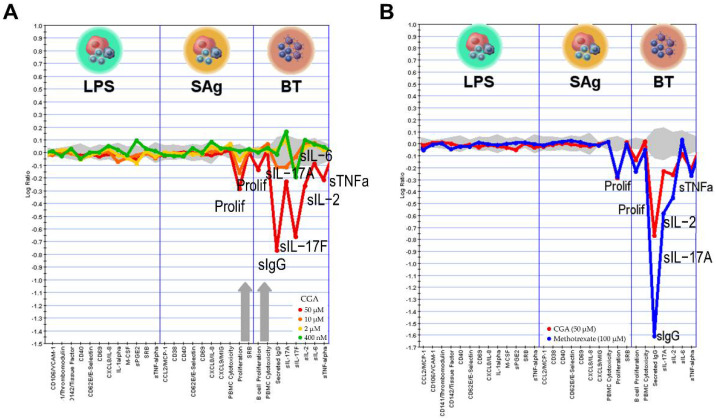
The biological activity of CGA was tested in 3 BioMAP systems at 4 concentrations (**A**) and overlays of CGA (50 μM) and methotrexate (MTX) (100 μM) (**B**). The readout proteins are shown along the *x*-axis. The grey region closest to the axis represents the range of historical vehicle control data. The annotated peaks constitute biomarker activities that show statistical significance when compared to historical vehicle controls after compound therapy.

We investigated the biological activity of CGA in BioMAP human primary cell co-culture systems. The panel covered both the potential off-target effects and the on-target effects by using a wide concentration range from 0.4 to 50 μM. The results revealed that CGA was found to be active in BioMAP Diversity PLUS with no obvious cytotoxicity. Figure 6 demonstrates that CGA had significant inhibitory effects on the T cell activation (SAg) system and the B and T cell autoimmunity (BT) system. The SAg system mimics vascular inflammation and T cell activation, in which human umbilical vein endothelial cells (HUVEC) were cultured with SAg and peripheral blood mononuclear cells (PBMC) [15]. The BT system simulates germinal center T cell dependent B cell activation and class switching [16]. In the BT system, anti-IgM and light TCR stimulation were used to the activate B cell and PBMC co-culture for 84 h. In addition, CGA significantly inhibited IL-17F release in the BT system, as well as IL-17A, IL-2, IL-6, and TNF-α, and secreted IgG at the concentration of 50 μM (Figure 6A). Then, we conducted an unsupervised search of the BioMAP reference database for biologically similar agents, including experimental chemical compounds and biological and approved drugs. Figure 6B indicates that the phenotypes induced by CGA most closely resembled those induced by the dihydrofolate reductase inhibitor methotrexate (MTX) (Pearson’s correlation, r = 0.895). As a result of methotrexate’s impairment of folic acid metabolism, DNA synthesis and cellular replication are both inhibited. Methotrexate has been licensed for the treatment of psoriasis and rheumatoid arthritis. It is utilized at greater doses as an anti-cancer agent and at lower concentrations as an anti-inflammatory agent. The key features shared by CGA and MTX include the inhibition of proliferation in the SAg and BT system and the downregulation of secreted IgG, IL-17, IL-2, and TNF-α.

## 3. Discussion

Seventy-three to eighty-five percent SLE patients have cutaneous lesions, which can emerge at any stage of the disease [17]. The skin damage of SLE, regarded as cutaneous lupus erythematosus (CLE), is characterized by immune imbalance [12]. Skin pathology in SLE shows massive inflammatory cell infiltration, including innate immune cells and B and T cells. Induced by a variety of pathogenic factors, abnormal immune cells produce a large number of autoantibodies and inflammatory cytokines, which eventually lead to skin damage. Increasing evidence suggests that infiltrating T and B cells in the dermis may play a key role in skin damage in SLE [18].

The therapeutic drugs for SLE are mainly focused on hormones, antimalarials, immunosuppressants, and nonsteroidal anti-inflammatory drugs (NSAIDs), but they cannot overcome the side effects associated with their use in clinical treatment, such as relapse and liver and kidney fibrosis. In recent years, promising biologic therapies have been achieved in the treatment of SLE. Among these, the belimumab antibody and rituximab are most often used in the clinic [19]. Rituximab, an anti-CD20 antibody, although it is included in the European and US recommendations for the treatment of refractory lupus nephritis, failed in the treatment in SLE randomized controlled trials (RCTs). Belimumab only modestly depletes B cells and has now become the first new drug in the last five decades to be approved by the European Medicines Agency (EMA) and the Food and Drug Administration (FDA) for the treatment of active non-renal lupus. Anifrolumab [20], a monoclonal antibody antagonist of the type 1 interferon receptor (IFNAR), and Telitacicept [21] (a fusion protein) continue to be assessed in clinical studies on SLE in various countries. New therapies targeting the T helper 17 (Th17), BAFF, and calcineurin pathways showed positive phase 2b trial results despite the limited evidence available supporting the use of biologic therapies in SLE.

Natural product chemistry is closely related to human health. Compared to syn-thetic drug molecules, natural products are more complex in structure, larger in molecular weight, more hydrophobic, and more rigid in molecules and are still the main source of marketed oral drugs, although they do not conform to the “Lipinski class of five principles of pharmacology” [22]. Active natural products of plant origin account for a significant portion of the currently marketed drugs, and artemisinin, butylphthalide, and bicyclic alcohol, which are currently in clinical use, are derived from plants. CGA is a polyphenolic compound with a variety sources, especially green coffee, honeysuckle, and eucommia leaves [23]. CGA can regulate the expression of inflammatory factors and relieve the organ damage caused by inflammation. Recent studies have demonstrated that CGA can inhibit oxide production and suppress T cell proliferation to exert anti-arthritic effects [24]. Shi et al. found that CGA may inhibit the Th17 differentiation to alleviate allergic rhinitis (AR)-induced behaviors [25]. In addition, CGA significantly decreased T cell counts and the elevation of Th2 cytokines, resulting in the suppression of Th1 cytokines [26]. Although various researchers have verified that CGA can modulate the immune system, whether and how CGA acts in autoimmune disease have rarely been reported. In this study, we explored the protective effects of CGA on MRL/lpr mice and the underlying mechanism associated with the cytokines.

Ten-week-old female MRL/lpr mice were treated with CGA or CTX for 3 months be-fore being sacrificed. CGA could significantly reduce the skin damage rate and alleviate the lupus skin mucosal damage. We observed the effect of CGA on the skin lesions under the light microscope and made pathological scores for the MRL/lpr and Balb/c mice. The skin lesions of the model group were more obvious and manifested as a thickened spinous cell layer, hyperkeratosis, hyperplasia of the dermal fibrous tissue, and the infiltration of inflammatory cells. Compared with the model group, the skin lesions in the CGA group were reduced to some extent. Subsequently, we determined the number of mast cells in the skin of different groups of mice. The number of mast cells in the dermis of the model group was significantly increased, while the average number of dermal mast cells in the CGA group was less. The number of mast cells in the dermis of the animals in the model group was significantly increased, while a certain degree of decrease was observed after the CGA treatment.

At the same time, we observed that CGA significantly alleviated joint swelling in the MRL/lpr mice. Pathology showed that the infiltration of capsulitis cells and cartilage erosion were seen in the model group, while the joint lesions in the CGA group were slightly alleviated. These results suggested that the alleviation effect of the SLE symptoms in the MRL/lpr mice by CGA may be systemic; so, we detected the levels of disease-related factors in the serum. CGA could reduce the dsDNA level while no significant change was observed in the ANA titers, which indicates that CGA did not have a significant activating effect on the B cells. Subsequently, we examined the levels of T cell-related cytokines, and the ELISA results showed that CGA significantly diminished the serum levels of IL-17A and IL-17F. The results of the BioMAP human primary cell co-culture systems also suggest that CGA can significantly reduce IL-17 levels in biologically relevant in vitro models of human autoimmune diseases.

SLE is generally considered to be associated with the polyclonal activation of B and T lymphocytes [27]. The imbalance between regulatory T cells (Tregs) and Th17 cells was found to play important roles in the pathogenesis of SLE [28]. Th17 cells can secrete large amounts of IL-17, a pro-inflammatory cytokine, in response to pro-inflammatory factors that contribute to the pathogenesis of many inflammatory diseases [29]. The IL-17 family consists of six members, including IL-17A, IL-17B, IL-17C, IL-17D, IL-17E, and IL-17F. Among them, IL-17A and IL-17F are highly homologous and can induce an increase in inflammatory factors upon binding to receptors, promoting the inflammatory response and ultimately causing severe tissue damage. In mouse models of lupus, the levels of IL-17 are significantly elevated, and the genetic deletion of IL-17 has been shown to improve the pathology of SLE. Moreover, the level of IL-17-producing cells was increased in the peripheral blood of SLE patients, as well as in target tissues such as the kidney [30]. IL-17 is expected to be a new target for SLE treatment.

We compared CGA with 4500 compounds in the BioMAP database and found that CGA has the most similar mechanism of action to MTX in the BT system. MTX can treat cancers such as breast and lung cancer by interfering with the biosynthesis of purines. Low-dose MTX therapy remains a standard-of-care treatment for psoriatic arthritis, rheumatoid arthritis, and other autoimmune diseases [31]. MTX can augment the sensitivity of T cells to apoptosis by inhibiting dihydrofolate reductase, which in turn dampens the immune response [32]. Th17 activity is believed to be decreased by regulatory T cells (Treg cells), a subpopulation of immunoregulatory cells that express the forkhead box protein P3 (FOXP3). MTX can enhance the generation of adenosine through FOXP3 T + reg cells to moderate the immunotolerant environment to ameliorate rheumatoid arthritis (RA) damage [33]. This suggests that CGA may exert organismal protective effects by modulating the immune microenvironment, and more research is needed to verify this.

## 4. Materials and Methods

### 4.1. Mice and Treatment

Female MRL/lpr mice were obtained from Shanghai SLAC Laboratory Animal Co., Ltd., at 9 weeks of age. Nine-week-old female Balb/c mice were obtained from Beijing Vital River Laboratory Animal Technology Co., Ltd. The mice were housed under specific pathogen-free conditions and given access to food and water. All the mice were acclimatized in the facility for 1 week prior to the experiments. The animal experiments were approved by the Institute of Materia Medica, Chinese Academy of Medical Sciences and Peking Union Medical College (approval number: 00003885).

We started the CGA intervention from the 10th week. The MRL/lpr mice were randomly assigned to one of three groups: model (*n* = 8, saline); CTX (*n* = 7, 50 mg/kg/week, i.p.); and CGA (*n* = 8, 40 mg/kg/day, i.p.). The Balb/c mice (*n* = 8, saline) were considered as the control group. The whole administration process lasted for 12 weeks. Serum was isolated from the mice at 14, 18, 20, and 22 weeks old. Then, the animals were sacrificed for follow-up research. The blood samples were left undisturbed at room temperature for 1–2 h and then collected into serum separation tubes and centrifuged at 3000–5000 rpm for 15 min at 4 °C. The serum samples obtained were stored at −80 °C until use.

### 4.2. Chemicals

Jiuzhang Biochemical Engineering Science and Technology Development Co., Ltd. (Chengdu, China) provided the CGA. Normal saline was used as a vehicle in this study; the CGA was dissolved in it at a concentration of 40 mg/kg. CTX, the positive drug in this study, was purchased from Baxter Oncology GmbH; it was dissolved with normal saline at a concentration of 50 mg/kg.

### 4.3. Pathology of Skin and Joint Tissues

HE staining and toluidine blue staining were performed with 5 μM sections. The skin tissue lesions were examined under the microscope, and SPSS 13.0 software was used for statistical analysis. The statistical methodology used the chi-square test.

### 4.4. Measurement of Serum Antibodies

The anti-nuclear antibody profile enzyme-linked immunosorbent assay (ANA ELISA) kit detects a highly purified autoantigen encapsulated in parallel on a dye strip. It is used for the in vitro qualitative detection of anti-Sm, PCNA, dsDNA, nucleosomes, histones, and ribosomal P proteins. Immediately following blood collection, the mice were euthanized; the serum was collected after centrifugation (2000× *g* for 10 min at 4 °C). Anti-dsDNA antibody levels were measured in 1:10,000 dilution of sera by using ELISA kits (Euroimmun, Lubeck, Germany) according to the manufacturer’s instructions. For autoantibody (ANA) staining, the serum was diluted with PBS (1:100); the procedure was performed according to the manufacturer’s instructions (Euroimmun, Lubeck, Germany).

### 4.5. Serum ELISA for Inflammatory Cytokines

The levels of mouse serum inflammatory cytokines (IL-17A, IL-17F, IL-6, IFN-γ IgA, IgM, IgG, BAFF and C3) were analyzed by commercial ELISA kits (Cloud-Clone Corp, Wuhan, China) following the manufacturer’s protocols. The calibration curves were used to calculate the results, which were expressed in pg/mL.

### 4.6. BioMAP Profiling Analysis

The BioMAP systems employed are shown in Table 2. The protocols for preparation and compound profiling were as previously described [34,35,36]. CGA was added 1 h before treatment and was presented for stimulation. Cell-based, enzyme-linked immunosorbent assays (ELISAs) were performed as previously described.

### 4.7. Statistical Analysis

The results are shown as mean ± SD. The statistical analysis of the data was performed using Graphpad 8.0 (GraphPad Software, San Diego, CA, USA). The differences between the experimental groups were revealed using one-way ANOVA with Dunnett’s multiple comparisons test. The control, CTX, and CGA groups were compared to the model group in the multiple comparison tests. *p* < 0.05 was considered statistically significant.

## 5. Conclusions

In conclusion, CGA ameliorated skin damage and arthritis in MRL/lpr mice. Furthermore, CGA attenuated serum anti-dsDNA to some extent. CGA significantly diminished IL-17 levels in both the serum and the human primary cells. Taken together, CGA could serve as a promising natural candidate for the treatment of SLE-mediated skin lesions and arthritis.

## Figures and Tables

**Table 1:** Pathological damage score of MRL/lpr mice.

	Acanthosis		Hyperkeratosis		Skin Mast Cells	Mast Cell Reduction Rate (%)
	Incidence *	Chi-Squared Statistic	Incidence #	Chi-Squared Statistic		
model	100% (0/8)		63% (5/8)		96.375	
CTX	29% (2/7)	*p* = 0.007	29% (2/7)	*p* = 0.315	24.4286	75%
CGA	29% (2/7)	*p* = 0.007	0 (0/3)	*p* = 0.026	47.8571	50%

* degree of acanthosis, none (0) to marked thickened dermis (2); [14]. # hyperkeratosis, none (0) to markedly increased keratin (2).

**Table 2:** The BioMAP systems utilized in compound profiling. Cell types are shown as cultured and stimulated for 72 h with environmental factors (added in addition to CGA).

System	Cell Type	Environment	Readouts
LPS 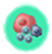	Peripheral blood mononuclear cells + Venular endothelial cells	TLR4	MCP-1, VCAM-1, TM, TF, CD40, E-selectin, CD69, IL-8, IL-1α, M-CSF, sPGE2, SRB, sTNFα
SAg 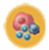	Peripheral blood mononuclear cells + Venular endothelial cells	Superantigens (TCR)	MCP-1, CD38, CD40, E-selectin, CD69, IL-8, MIG, PBMC Cytotoxicity, Proliferation, SRB
BT 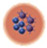	Peripheral blood mononuclear cells + B cells	Anti-IgM, TCR	B cell Proliferation, PBMC Cytotoxicity, Secreted IgG, sIL-17A, sIL-17F, sIL-2, sIL-6, sTNFα

## Data Availability

Data is contained within the article and Supplementary Material.

## Supplementary Materials

The following supporting information can be downloaded at: https://www.mdpi.com/article/10.3390/ph15111327/s1, Figure S1: the effects of CGA on serum cytokines in serum. CGA had no significant effect on serum IgA, IgG, IgM, BAFF, and C3.
