# Supplementary material for: Chlorogenic Acid Relieves the Lupus Erythematosus-like Skin Lesions and Arthritis in MRL/lpr Mice
Source: Pharmaceuticals (Basel). 2022 Oct 27;15(11):1327. doi: 10.3390/ph15111327 (PMC9697989; doi:10.3390/ph15111327)
Supplement: Supplementary file 1 [file pharmaceuticals-15-01327-s001.zip › pharmaceuticals-1982323-supplementary.pdf]

Chlorogenic acid relieves the lupus erythematosus-like skin lesions and arthritis in MRL/lpr mice

Ruxuan Wang [1,†], Xiaoyi Yang [2,†], Shen You [1], Mengyao Hao [1], Jianguang Li [3,*], Xiaoguang Chen [1,*] and Jing Jin [1,*]

[1] State Key Laboratory of Bioactive Substance and Function of Natural Medicines, Institute of Materia Medica, Chinese Academy of Medical Sciences & Peking Union Medical College, Beijing 100050, China
[2] College of Pharmacy, Xinjiang Medical University, Urumqi 830054, China
[3] College of Pharmacy, Xinjiang University of Science and Technology, Korla 841000, China
* Correspondence: lijianguang@xjust.edu.cn (J.L.); chxg@imm.ac.cn (X.C.); rebeccagold@imm.ac.cn (J.J.); Tel.:+86-0996-8871835 (J.L.); +86-10-63165207 (X.C. & J.J.)
† These authors contributed equally to this work.

Results

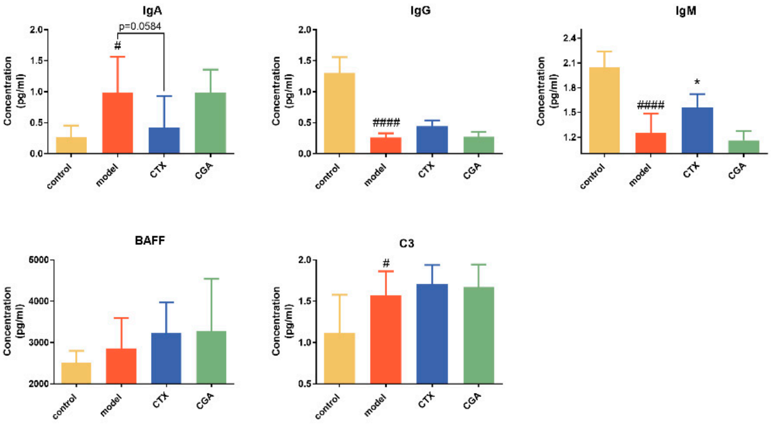

Figure S1 The effects of CGA on serum cytokines in serum. CGA had no significant effect on serum immunoglobulin A (IgA), immunoglobulin G (IgG), immunoglobulin M (IgM) , B-cell Activating factor of the TNF family (BAFF) and complement 3 (C3).
